# Randomised clinical trial of ferric citrate hydrate on anaemia management in haemodialysis patients with hyperphosphataemia: ASTRIO study

**DOI:** 10.1038/s41598-019-45335-4

**Published:** 2019-06-20

**Authors:** Keitaro Yokoyama, Masafumi Fukagawa, Takashi Akiba, Masaaki Nakayama, Kyoko Ito, Koji Hanaki, Myles Wolf, Hideki Hirakata

**Affiliations:** 1 Division of Nephrology and Hypertension, Department of Internal Medicine, Jikei University School of Medicine, Tokyo, Japan; 2 Division of Nephrology, Endocrinology and Metabolism, Tokai University School of Medicine, Kanagawa, Japan; 3 Tokyo Next Medical & Hemodialysis Clinic, Tokyo, Japan; 4 Tohoku University Hospital, Miyagi, Japan; 5 St. Luke’s International University, St. Luke’s International Hospital, Tokyo, Japan; 6 Medical Affairs Department, Torii Pharmaceutical Co. Ltd., Tokyo, Japan; 7 Pharmaceutical Division, Japan Tobacco Inc., Tokyo, Japan; 8 Division of Nephrology, Department of Medicine, and Duke Clinical Research Institute, Duke University School of Medicine, Durham, NC USA; 9 Fukuoka Renal Clinic, Fukuoka, Japan

**Keywords:** End-stage renal disease, Phosphorus metabolism disorders

## Abstract

Ferric citrate hydrate (FC) is an iron-based phosphate binder approved for hyperphosphataemia in patients with chronic kidney disease. We conducted a randomised controlled trial to evaluate the effects of FC on anaemia management in haemodialysis patients with hyperphosphataemia. We 1:1 randomised 93 patients who were undergoing haemodialysis and being treated with non-iron-based phosphate binders and erythropoiesis-stimulating agents (ESA) to receive 24 weeks of FC or to continue their non-iron-based phosphate binders (control) in a multicentre, open-label, parallel-design. Phosphate level was controlled within target range (3.5–6.0 mg/dL). The primary endpoint was change in ESA dose from baseline to end of treatment. Secondary endpoints were changes in red blood cell, iron and mineral, and bone-related parameters. Compared with control, FC reduced ESA dose [mean change (SD), −1211.8 (3609.5) versus +1195 (6662.8) IU/week; P = 0.03] without significant differences in haemoglobin. FC decreased red blood cell distribution width (RDW) compared with control. While there were no changes in serum phosphate, FC reduced C-terminal fibroblast growth factor (FGF) 23 compared with control. The incidence of adverse events did not differ significantly between groups. Despite unchanged phosphate and haemoglobin levels, FC reduced ESA dose, RDW, and C-terminal FGF23 compared with control.

## Introduction

In patients undergoing haemodialysis, hyperphosphataemia and anaemia are complex management challenges^1,2^. Hyperphosphataemia promotes vascular calcification and is associated with increased risk of mortality^3,4^. Anaemia promotes progression of left ventricular hypertrophy and increases risk of heart failure^5^. When used to treat renal anaemia, erythropoiesis-stimulating agents (ESA) improves quality of life^6^, and reduces the number of patients requiring blood transfusions; however, some patients respond insufficiently to ESA treatment. Administration of high-dose ESA to these patients can worsen their clinical outcomes^7^. Iron deficiency is an important contributor to anaemia in patients undergoing haemodialysis and to their insufficient therapeutic responses to ESA^8–10^. Maintaining iron sufficiency is thus critical for adequate treatment of renal anaemia.

Clinical studies of ferric citrate (Auryxia®, Keryx Biopharmaceuticals, Inc., Boston, USA) in patients with hyperphosphataemia undergoing maintenance haemodialysis showed not only reductions in serum phosphate, but also reductions in doses of ESA and intravenous (IV) iron, likely because of increased iron absorption during ferric citrate administration. These studies were conducted in the United States and Israel, where ESA and IV iron were used by approximately 80% of trial participants^11,12^. Rates of ESA utilisation are similar in Japan, but IV iron use is far less common and serum ferritin levels are far lower in Japanese patients undergoing haemodialysis^13^, demonstrating important regional differences in clinical characteristics and treatment of patients undergoing haemodialysis.

Given these differences, we conducted the ASTRIO Study [A Study examining The contribution to Renal anaemia treatment with ferric citrate hydrate (FC, Riona®, Torii Pharmaceutical Co. Ltd., Tokyo, Japan), Iron-based Oral phosphate binder], which is the first randomised, prospective, multicentre study specifically designed to investigate the effects of FC on treatment of renal anaemia in patients with hyperphosphataemia undergoing haemodialysis. We tested the hypothesis that treatment with FC would provide comparable control of hyperphosphataemia while simultaneously providing superior treatment of renal anaemia compared with non-iron-based phosphate binders in patients with hyperphosphataemia and anaemia who were undergoing haemodialysis and ESA therapy.

## Results

### Patient disposition and baseline characteristics

**Figure 1:**
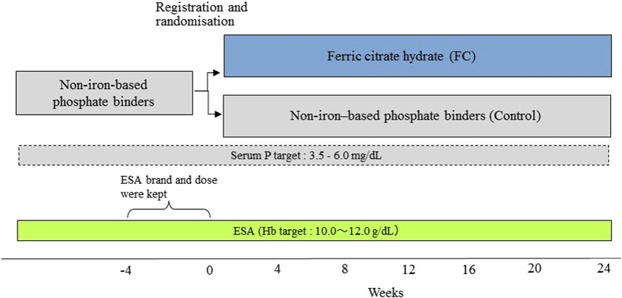
Study design.

**Figure 2:**
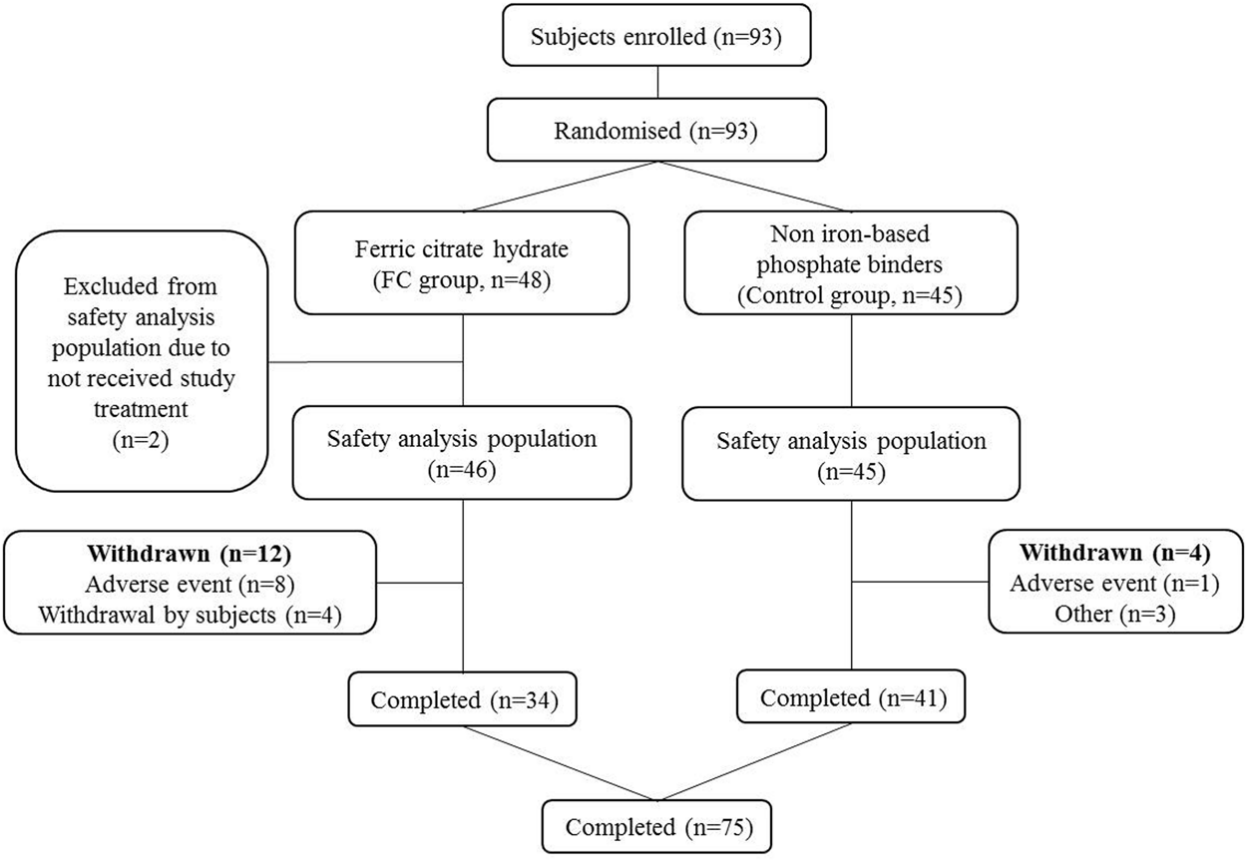
Consolidated standards of the reporting trial.

A total of 93 patients were randomised to the FC group (*n* = 48) or control group (*n* = 45). After excluding 2 patients who withdrew consent before receiving any FC, 91 patients were included in the efficacy analysis dataset. Across the two groups, 75 patients (82%) completed the full 24-week course of treatment (Figs 1 and 2). The proportion of discontinuations because of adverse events was higher in the FC (*n* = 8) versus the control group (*n* = 1), Treatment-related adverse events leading to withdrawal of FC included diarrhoea and pruritus (Day 7), constipation (Day 7), nausea (Day 17) and rectal tenesmus (Day 49). No treatment-related adverse event leading to withdrawal occurred in the control group. The mean FC dose during the study period was 1610 mg/day (containing approximately 386 mg ferric iron). There were no statistically significant differences in demographics or baseline characteristics between the two groups (Table 1). The proportion of diabetic nephropathy as the main underlying disease leading to haemodialysis trended lower in the FC group than the control group (P = 0.09). There were no differences in the mean (SD) ESA dose and the mean (SD) C-reactive protein (CRP) between the groups at baseline.

**Table 1:** Baseline characteristics.

Variables	FC (*n* = 46)	Control (*n* = 45)	P-value
Age (yr), mean (SD)	63.3 ± 10.0	62.7 ± 12.7	0.78^a^
Body weight before dialysis (kg)	60.02 ± 10.66	62.91 ± 13.61	0.26^a^
Sex (male), *n* (%)	30 (65.2%)	36 (80.0%)	0.16^b^
Main underlying disease, *n* (%)			
Chronic glomerulonephritis	15 (32.6%)	11 (24.4%)	0.49^b^
Diabetic nephropathy	19 (41.3%)	27 (60.0%)	0.09^b^
Nephrosclerosis	7 (15.2%)	3 (6.7%)	0.32^b^
History of haemodialysis ≥1 year, *n* (%)	43 (93.5%)	40 (88.9%)	0.49^b^
Phosphate binders, *n* (%)^c^			
Calcium carbonate	30 (65.2%)	28 (62.2%)	0.83^b^
Sevelamer hydrochloride	6 (13.0%)	10 (22.2%)	0.28^b^
Bixalomer	4 (8.7%)	5 (11.1%)	0.74^b^
Lanthanum carbonate hydrate	21 (45.7%)	21 (46.7%)	1.00^b^
ESA, *n* (%)			
Epoetin alpha	21 (45.7%)	14 (31.1%)	0.20^b^
Epoetin beta	1 (2.2%)	3 (6.7%)	0.36^b^
Darbepoetin alpha	17 (37.0%)	21 (46.7%)	0.40^b^
Epoetin beta pegol	7 (15.2%)	7 (15.6%)	1.00^b^
Intravenous iron preparations (yes), *n* (%)	4 (8.7%)	6 (13.3%)	0.52^b^
Serum P (mg/dL), mean (SD)	5.36 (1.15)	5.15 (1.25)	0.42^a^
Serum Ca (mg/dL) (adjusted), mean (SD)	9.42 (0.61)	9.50 (0.70)	0.52^a^
i-PTH (pg/mL), mean (SD)	116.2 (79.7)	154.8 (120.0)	0.07^a^
Hb (g/dL), mean (SD)	10.52 (0.70)	10.47 (0.94)	0.78^a^
TSAT (%), mean (SD)	23.0 (9.8)	21.2 (9.3)	0.36^a^
Serum ferritin (ng/mL), mean (SD)	105.7 (85.5)	85.6 (85.8)	0.27^a^
Hepcidin-25 (ng/mL), mean (SD)	59.0 (50.1)	44.2 (46.5)	0.15^a^
ESA dose^d^ (IU/week), mean (SD)	5735.4 (4933.3)	5848.1 (4082.8)	0.91^a^
ERI, mean (SD)	9.81 (9.26)	9.99 (7.53)	0.92^a^
MCV (fL), mean (SD)	94.1 (5.3)	93.8 (7.0)	0.83^a^
MCH (pg), mean (SD)	30.80 (2.23)	30.56 (2.52)	0.63^a^
RDW (%), mean (SD)	14.85 (1.56)	15.38 (1.80)	0.14^a^
i-FGF23 (pg/mL), mean (SD)	11774.5 (14561.0)	7883.1 (10243.8)	0.14^a^
c-FGF23^e^ (pg/mL), mean (SD)	1610.6 (2370.9)	1185.8 (1608.6)	0.32^a^
Alpha-Klotho (pg/mL), mean (SD)	400.2 (107.1)	442.8 (239.1)	0.27^a^

FC, ferric citrate hydrate; SD, standard deviation; ESA, erythropoiesis-stimulating agent; P, phosphate; Ca, calcium; PTH, parathyroid hormone; Hb, haemoglobin; TSAT, transferrin saturation; ERI, erythropoiesis resistance index; MCV, mean corpuscular volume; MCH, mean corpuscular haemoglobin; RDW, red blood cell distribution width; FGF23, fibroblast growth factor 23.

^a^Student’s *t*-test.

^b^Fisher’s exact test.

^c^Numbers do not add up to 100% due to combination therapy.

^d^Epoetin 200 IU = darbepoetin 1 µg = epoetin beta pegol 1 µg.

^e^c-FGF23:1 pg/mL = 0.133 pmol/L.

### Anaemia management parameters

**Figure 3:**
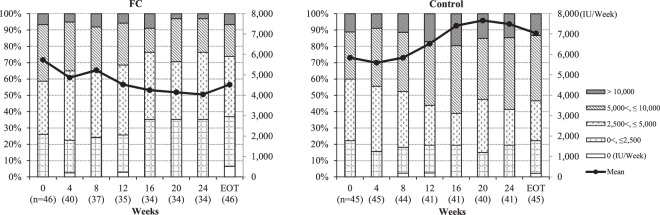
FC reduced ESA dose during the study. The proportions of patients receiving an ESA dose greater than 5,000 IU/week decreased in the FC group compared with the control [FC: baseline, n = 19, 41.3%; week 24, n = 8, 23.5%; control: baseline, n = 18, 40.0%; week 24, n = 24, 58.5%].

Haemoglobin (Hb) and haematocrit (Hct) were not changed in both groups. The mean (SD) ESA dose (IU/week) decreased from baseline to end of treatment (EOT) in the FC group (5735.4 [4933.3] to 4523.6 [4844.3]), but increased in the control group during the same period (from 5484.1 [4082.8] at baseline to 7039.6 [6830.8] at EOT). For the primary endpoint, the mean (SD) change in ESA dose was −1211.8 (3609.5) IU/week in the FC group versus +1195 (6662.8) IU/week in the control group (P = 0.03), and the change in ERI was −2.43 (5.90) versus +2.62 (13.56), respectively (P = 0.02). The mean (SD) cumulative dose of ESA was significantly lower in the FC versus the control group (P = 0.02; Table 2), and the proportion of patients receiving an ESA dose greater than 5,000 IU/week decreased in the FC versus the control group (Fig. 3).

**Table 2:** ESA dose, ERI, cumulative doses of ESA and intravenous iron, and biomarkers.

ESA dose and ERI from baseline to EOT								
Variables	FC (*n* = 46) Mean (SD)			Control (*n* = 45) Mean (SD)			Adjusted mean difference^a^	P-value^b^
	Baseline	EOT	Change	Baseline	EOT	Change		
ESA dose (IU/week)	5735.4 (4933.3)	4523.6 (4844.3)	−1211.8 (3609.5)	5848.1 (4082.8)	7039.6 (6830.8)	1191.5 (6662.8)	−2442.1	0.03
ERI	9.81 (9.26)	7.74 (8.35)	−2.43 (5.90)^d^	9.99 (7.53)	12.97 (13.79)	2.62 (13.56)^e^	−5.12	0.02
**Cumulative doses of ESA and intravenous iron preparation from baseline to Week 24**								
**Variables**	**FC (*n*** = **33)** **Mean (SD)**			**Control (*n*** = **40)** **Mean (SD)**				**P-value** ^**c**^
Cumulative dose of ESA (IU)	104431.8 (62639.5)			162400.0 (108519.2)				0.02
Cumulative dose of intravenous iron (mg)	105.5 (199.5)			330.0 (355.7)				0.01
**Biomarkers from baseline to EOT**								
**Variables**	**FC (*n*** = 4**0)** **Mean (SD)**			**Control (*n*** = 4**2)** **Mean (SD)**			**Adjusted mean difference** ^**a**^	**P-value** ^**b**^
	**Baseline**	**EOT**	**Change**	**Baseline**	**EOT**	**Change**		
Serum P (mg/dL)	5.36 (1.15)	5.65 (1.39)	0.24 (1.59)	5.15 (1.25)	5.04 (1.32)	−0.17 (1.53)	0.55	0.06
Serum Ca (mg/dL) (adjusted)	9.42 (0.61)	9.30 (0.59)	−0.08 (0.53)	9.50 (0.70)	9.67 (0.65)	0.16 (0.71)	−0.31	0.01
i-PTH (pg/mL)	116.2 (79.7)	141.7 (102.8)	30.4 (97.0)	154.8 (120.0)	138.2 (123.8)	−16.3 (86.9)	34.4	0.09
Hb (g/dL)	10.52 (0.70)	10.90 (1.23)	0.45 (1.33)	10.47 (0.94)	10.74 (1.14)	0.34 (1.73)	0.17	0.51
Ht (%)	32.20 (2.52)	32.90 (3.88)	0.94 (4.10)	32.14 (2.91)	32.83 (3.58)	0.88 (5.56)	0.07	0.94
RBC count (10^4^ µL)	343.6 (36.8)	347.1 (49.6)	5.7 (39.3)	344.6 (41.5)	364.2 (55.1)	20.1 (71.4)	−16.3	0.16
TSAT (%)	23.0 (9.8)	31.8 (13.6)	8.6 (12.1)	21.2 (9.3)	21.8 (10.8)	0.5 (11.8)	9.0	<0.001
Serum ferritin (ng/mL)	105.7 (85.5)	181.2 (108.2)	79.0 (81.5)	85.6 (85.8)	89.0 (97.4)	2.9 (79.3)	79.5	<0.001
Hepcidin-25 (ng/mL)	59.0 (50.1)	118.1 (86.2)	59.9 (72.9)	44.2 (46.5)	47.7 (60.3)	4.5 (48.0)	57.1	<0.001
MCV (fL)	94.1 (5.3)	95.2 (5.2)	1.2 (3.4)	93.8 (7.0)	90.9 (6.9)	−2.6 (4.5)	3.9	<0.001
MCH (pg)	30.8 (2.23)	31.57 (2.17)	0.76 (1.42)	30.56 (2.52)	29.76 (2.65)	−0.66 (1.88)	1.51	<0.001
RDW (%)	14.85 (1.56)	15.15 (1.47)	0.19 (1.59)	15.38 (1.80)	16.26 (2.43)	0.83 (2.11)	−0.83	0.04
i-FGF23 (pg/mL)^f^	8.5 (1.5)	8.4 (1.5)	−0.1 (0.8)	8.1 (1.5)	8.3 (1.5)	0.1 (0.9)	0.8^g^	0.33
c-FGF23 (pg/mL)^f^	6.6 (1.3)	6.3 (1.5)	−0.2 (0.8)	6.3 (1.3)	6.5 (1.3)	0.2 (0.8)	0.7^g^	0.04
Alpha-klotho (pg/mL)	400.2 (107.1)	399.7 (129.3)	2.0 (91.5)	442.8 (239.1)	440.3 (153.5)	−8.9 (145.3)	−11.1	0.58

Withdrawn (FC [n = 12] and control [n = 4]) and missing data at Week 24 (FC [n = 1] and control [n = 1]) are not included in the numbers.

ESA, erythropoiesis-stimulating agent; ERI, erythropoiesis resistance index; EOT, end of treatment (day of observation at week 24 or discontinuation); FC, ferric citrate hydrate; SD, standard deviation; P, phosphate; Ca, calcium; i-PTH, intact parathyroid hormone; Hb, haemoglobin; Ht, haematocrit; RBC, red blood cell; TSAT, transferrin saturation; MCV, mean corpuscular volume; MCH, mean corpuscular haemoglobin; RDW, red blood cell distribution width; FGF23, fibroblast growth factor 23.

^a^Adjusted mean difference: FC–Control.

^b^Ancova (covariate: baseline).

^c^Wilcoxon rank sum test.

^d^*n* = 40, ^e^*n* = 41.

^f^Logarithmic transformation.

^g^Exponential form of logarithmic adjusted mean difference.

### Red blood cell parameters

There were no statistically significant differences in the changes in Hb, Hct, or red blood cell count between the groups. However, the changes in mean corpuscular volume (MCV), mean corpuscular haemoglobin (MCH), and red cell distribution width (RDW) did differ significantly between groups (Table 2).

Patients with high RDW (≥15.5%) tended to have lower serum ferritin and higher ESA doses at baseline than patients with low RDW (<15.5%; Table 3). Within the high RDW stratum, FC induced a significant reduction in RDW compared with control, and a significantly larger reduction in ESA dose (P = 0.04). In the low RDW stratum, ESA dose tended to increase in the control versus the FC group, but the difference did not reach significance (P = 0.09; Table 3).

**Table 3:** Stratified analysis by baseline RDW.

Variables	Low RDW (<15.5%) at baseline							High RDW ( ≥ 15.5%) at baseline						
	FC Mean (SD)			Control Mean (SD)			P-value^a^	FC Mean (SD)			Control Mean (SD)			P-value^a^
	Baseline (*n* = 33)	Week 24 (*n* = 24)	Change	Baseline (*n* = 27)	Week 24 (*n* = 24)	Change		Baseline (*n* = 13)	Week 24 (*n* = 9)	Change	Baseline (n = 18)	Week 24 (*n* = 16)	Change	
RDW (%)	14.04 (0.76)	14.79 (1.09)	0.71 (0.97)	14.21 (0.73)	15.73 (1.98)	1.45 (1.65)	0.29	16.93 (1.01)	15.08 (1.17)	−1.76 (1.78)	17.13 (1.45)	17.21 (2.87)	−0.08 (2.55)	0.04
Hb (g/dL)	10.43 (0.69)	10.92 (1.22)	0.61 (1.24)	10.47 (0.87)	10.74 (1.15)	0.4 (1.56)	0.74	10.73 (0.71)	10.93 (1.09)	0.31 (1.18)	10.47 (1.05)	10.79 (1.19)	0.36 (2.05)	0.61
Serum ferritin (ng/mL)	117.82 (86.49)	200.29 (108.12)	91.42 (86.07)	100.10 (100.45)	84.91 (94.58)	−18.5 (58.91)	<0.001	75.04 (77.71)	147.64 (84.40)	57.59 (73.38)	63.89 (52.99)	88.81 (105.45)	23.58 (96.63)	0.18
TSAT (%)	25.12 (9.65)	36.0 (14.39)	10.50 (12.50)	21.56 (8.14)	20.0 (7.51)	−2.0 (11.14)	<0.001	17.77 (8.52)	27.11 (9.74)	9.0 (11.93)	20.61 (11.07)	23.94 (14.54)	3.94 (12.63)	0.31
ESA dose (IU/week)	4571.58 (4144.67)	3921.88 (2567.52)	−533.6 (3792.7)	4208.49 (2504.61)	7179.51 (7662.88)	2869.9 (7401.7)	0.09	8689.66 (5681.18)	4330.00 (2624.16)	−2887.2 (2318.3)	8307.47 (4782.88)	7947.79 (6074.62)	−571.5 (5822.6)	0.04
ERI	7.60 (6.53)	6.25 (3.69)	−1.11 (6.01)	7.27 (4.89)	12.53 (15.13)	5.11 (14.57)	0.05	15.43 (12.67)	7.81 (4.84)	−4.60 (4.70)	14.06 (8.99)	13.88 (12.40)	−1.01 (11.86)	0.26

RDW, red blood cell distribution width; FC, ferric citrate hydrate; SD, standard deviation; Hb, haemoglobin; TSAT, transferrin saturation; ESA, erythropoiesis-stimulating agent; ERI, erythropoiesis resistance index.

^a^Wilcoxon rank sum test.

### Iron-related parameters

In total, 15 patients (32.6%) in the FC group and 25 patients (55.6%) in the control group received IV iron (Fesin®, Saccharated Ferric Oxide, Nichi-Iko Pharmaceutical Co., Ltd., Toyama, Japan; the only approved IV iron in Japan) during the study. The mean (SD) cumulative doses of IV iron from baseline through week 24 was significantly lower in the FC versus the control group (105.5 [199.5] mg versus 330.0 [355.7] mg; P = 0.01; Table 2).

**Figure 4:**
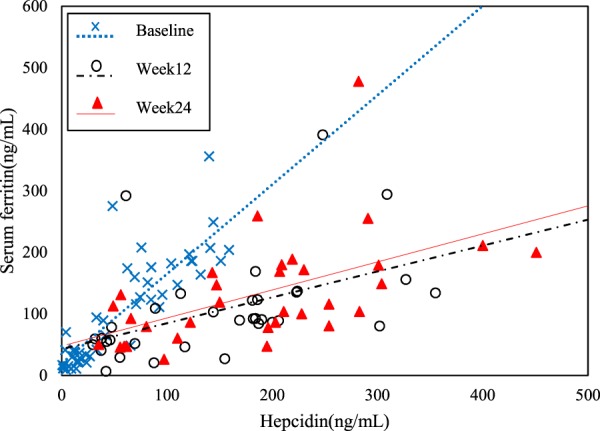
Scatter plot of serum ferritin and hepcidin in the FC group. The actual measured values obtained at baseline, weeks 12 and 24 were subjected to regression analyses with serum ferritin as the objective variable and hepcidin as the explanatory variable to calculate Pearson’s correlation coefficients and P-values. Baseline (n = 46): y = 19.99 + 1.45x; *r* = 0.85 (P < 0.001). Week 12 (n = 34): y = 90.05 + 0.56x; *r* = 0.48 (P < 0.004). Week 24 (n = 33): y = 100.81 + 0.64x; *r* = 0.54 (P < 0.001).

The mean (SD) serum ferritin increased significantly from baseline to EOT in the FC group (105.7 [85.5] ng/mL to 181.2 [108.2] ng/mL), but there was no significant change in the control group during the same period (85.6 [85.8] ng/mL to 89.0 [97.4] ng/mL). The mean (SD) hepcidin also increased significantly from baseline to EOT in the FC group (59.0 [50.1] ng/mL to 118.1 [86.2] ng/mL), but did not significantly change in the control group during the same period (44.2 [46.5] ng/mL to 47.7 [60.3] ng/mL). There were no significant differences between groups in mean (SD) levels of CRP [FC: baseline, 0.31 (0.61); week 24, 0.17 (0.17) mg/dL; control: baseline, 0.23 (0.44); week 24, 0.42 (1.13) mg/dL] or albumin [FC: baseline, 3.68 (0.32); week 24, 3.66 (0.37) g/dL; control: baseline, 3.68 (0.42); week 24, 3.55 (0.45) g/dL] throughout the study period. When the correlation between serum ferritin and hepcidin was examined at baseline and at weeks 12 and 24, the correlation changed from r = 0.85 (P < 0.001) at baseline to r = 0.48 (P < 0.004) at week 12 and r = 0.54 (P* < *0.001) at week 24, and the slope of the regression line also changed over time (Fig. 4).

### Chronic kidney disease (CKD) /Mineral bone disorder (MBD) parameters

There were no statistically significant differences in changes in serum phosphate [mean change (SD): FC, +0.24 (1.59) versus control, −0.17 (1.53); P = 0.06], intact-parathyroid hormone (i-PTH), α-klotho, or intact fibroblast growth factor (FGF) 23 between the two groups (Table 2). However, the FC group had significantly reduced C-terminal FGF23 levels compared with the control group (P = 0.04).

### Safety

Adverse events reported by ≥5% of patients in either group are shown in Table 4. Adverse events occurred in 32 patients (69.9%) in the FC group versus 30 patients (66.7%) in the control group (P = 0.82). The most common adverse events were diarrhoea in 7 patients (15.2%) in the FC group and nasopharyngitis in 7 patients (15.6%) in the control group. No treatment-related serious adverse events occurred in either group.

**Table 4:** Summary of safety.

Adverse Events	FC (*n* = 46)		Control (*n* = 45)	
	Number of AEs	Number of subjects (%)	Number of AEs	Number of subjects (%)
**AEs reported at ≥5% in either group**				
Total^a^	75	32 (69.6)	72	30 (66.7)
Diarrhoea	7	7 (15.2)	1	1 (2.2)
Nasopharyngitis	5	5 (10.9)	7	7 (15.6)
Back pain	2	2 (4.3)	7	7 (15.6)
Shunt stenosis	3	3 (6.5)	1	1 (2.2)
Influenza	1	1 (2.2)	3	3 (6.7)
Pharyngitis	1	1 (2.2)	4	4 (8.9)
Pruritus	1	1 (2.2)	3	3 (6.7)

AE, adverse event; FC, ferric citrate hydrate.

^a^Fisher’s exact test P = 0.82.

## Discussion

The prospective, multicentre ASTRIO study compared the effects of FC versus non-iron-based phosphate binders on anaemia management among Japanese patients who were undergoing maintenance haemodialysis and were also receiving phosphate binders for treatment of hyperphosphataemia and ESA for treatment of anaemia. While control of serum phosphate and Hb concentrations were comparably maintained within their reference ranges in the FC and control groups according to the clinical practice guidelines issued by the Japanese Society for Dialysis Therapy (JSDT), FC significantly decreased ESA and IV iron doses, RDW and C-terminal FGF23, and significantly increased MCV and MCH compared to control without significant increases in the incidence of adverse events (Table 4). These results demonstrate that management of hyperphosphataemia using FC can simultaneously enhance key aspects of anaemia management and reduce utilisation of concomitant anaemia treatments such as ESA and IV iron. Our findings align with previous results that suggested that haematopoiesis remained well supported during treatment with FC^12^.

During the study period, Hb was controlled within its reference range in both groups, but the ESA dose decreased in the FC group over time (Fig. 3). This finding is likely attributable to enhanced iron absorption during FC administration that was transported to the bone marrow to be utilised for erythropoiesis. The significant differences in the changes in erythrocyte parameters MCV and MCH support a beneficial effect of FC on iron utilisation for erythropoiesis (Table 2). A previous study showed that MCV elevation correlated with the change in Hb following administration of an oral iron preparation in iron-deficient haemodialysis patients^14^. These data suggest that a portion of the iron absorbed during FC administration was utilised for Hb production and led to production of mature erythrocytes with higher Hb concentrations. In addition, a significant difference in the change in RDW was found between the two groups (Table 2). A previous study reported that RDW decreased significantly after administration of an oral iron preparation in iron-deficient children^15^ further supports enhanced iron absorption and utilisation during FC administration as a mechanism that promoted normalisation of erythropoiesis, marked by relatively decreased RDW.

Increased RDW is associated with higher risk of mortality in the general population^16^ and in patients undergoing haemodialysis with RDW ≥15.5%^17^. In the present study, patients with higher RDW (≥15.5%) tended to have lower serum ferritin and higher ESA doses at baseline (Table 3). Among patients with higher RDW, those treated with FC experienced greater reductions in RDW and ESA dose, which suggests that this group absorbed a larger fraction of iron during FC administration. Since higher RDW might be a biomarker for greater bone marrow need for iron, these results suggest that FC is especially effective at enhancing anaemia management, restoring effective utilisation of ESA and thus, lowering ESA requirements in higher risk patients with higher RDW.

The relevant practice guideline in Japan recommends a trial of IV or oral iron in patients with CKD on ESA therapy with Hb <10 g/dL, serum ferritin <100 ng/mL, and TSAT <20%. As in previous studies of Japanese haemodialysis populations^13^, IV iron use in this study was low. Nevertheless, we confirmed that FC significantly decreased IV iron dose among patients who required IV iron. Our findings also support the previous suggestion that continued use of FC can provide a source of maintenance iron, reducing or eliminating the need for IV iron^12^. In the FC group, serum ferritin and hepcidin increased significantly compared with the control group (Table 2). Hepcidin is the main regulator of iron absorption from the small intestine^14^. In addition, hepcidin concentration increases in the settings of increased plasma iron concentrations and inflammation^18^. As the levels of CRP remained constant during the study period and the incidence of infections were not higher in the FC versus the control group, inflammation is unlikely to have caused the elevation of hepcidin. Instead, the FC group’s rise in serum ferritin and hepcidin levels is likely to be primarily a consequence of increased oral iron absorption.

Previous studies reported that hepcidin is produced in response to stored iron in blood and cells^19–22^. When checking the correlation between serum ferritin and hepcidin in the FC group at baseline and at weeks 12 and 24, the slopes of the regression line for serum ferritin and hepcidin were flatter at weeks 12 and 24 than at baseline (Fig. 4). While this suggests that hepcidin increased as negative feedback to suppress further iron absorption from the small intestine, confirmation of this result, which is based on clinical correlation, will require further investigation.

In the present study, patients already taking non-iron-based phosphate binders were randomised. Accordingly, the levels of serum phosphate were already controlled at baseline. There was no statistically significant difference in the change in serum phosphate between the two groups throughout the 24-week study period, while C-terminal FGF23 decreased in the FC group compared with the control group (Table 2). In CKD patients, FGF23 production is stimulated by disease progression, inflammation, and iron deficiency^23^. As such, CKD patients were reported to have elevated intact FGF23 and C-terminal FGF23 because of increased FGF23 production and relatively decreased FGF23 cleavage, with C-terminal FGF23 being increased more markedly^24,25^. The reduction in C-terminal FGF23 that we observed was likely attributable to repletion of iron stores by FC that reduced FGF23 synthesis and hence the requisite cleavage that generates the C-terminal peptide fragments that only the C-terminal FGF23 assay recognises. Unlike previous studies in which administration of FC for 12 or 24 weeks to patients with more severe baseline iron deficiency also decreased intact FGF23^26,27^, the lack of intact FGF23 reduction in the FC group in the present study might be attributable to the less severe iron deficiency at baseline in the study.

There are several limitations to the present study. First, the number of patients was relatively small and the study duration relatively short. In addition, a higher discontinuation rate due to AEs occurred in the FC versus the control group (8 patients vs. 1; Fig. 2). It may reduce the potency of this study on several variables. Second, patients in the control group continued to take the phosphate binders that they were using before registration, whereas in the FC group, non-iron-based phosphate binders were switched to FC at registration. This difference may have influenced the higher incidence rate of diarrhoea and other AEs that led to withdrawal in the FC group. Third, the lack of placebo-control could have affected the results that were based on physician judgement. Unfortunately, it is ethically challenging to conduct placebo-controlled studies of phosphate binders in patients with end stage renal disease.

In conclusion, treatment of hyperphosphataemia with FC simultaneously promoted erythropoiesis via partially absorbed iron, and thus, could enhance treatment of renal anaemia in Japanese patients with hyperphosphataemia undergoing maintenance haemodialysis.

## Methods

### Study design

This randomised, open-label, active-controlled, multicentre, parallel-arm, 24-week study was conducted at 17 centres in Japan from November of 2015 to January 2017 (UMIN000019176, registered on October 1, 2015). The study was performed in accordance with the Declaration of Helsinki and the Ethical Guidelines for Medical and Health Research Involving Human Subjects (dated 22 December 2014, Ministry of Health, Labour and Welfare in Japan). The study protocol was approved by an independent ethics committee and institutional review board of The Jikei University School of Medicine. All study participants provided written informed consent before undergoing any study procedures.

### Study population

The key inclusion criteria included adult patients aged ≥20 years being treated with maintenance haemodialysis consistently for at least 12 weeks before registration, who were receiving treatment with non-iron-based phosphate binders for hyperphosphataemia for at least 4 weeks before registration, and who were receiving ESA therapy for renal anaemia with unchanged ESA brand and constant dose for at least 4 weeks before registration. If the most recent ESA treatment interval before registration exceeded 2 weeks, patients were to have remained on a constant dose for a period twice as long as the most recent treatment interval.

The key exclusion criteria were hypersensitivity to any ingredient of FC; gastrointestinal diseases such as peptic ulcer or inflammatory bowel disease; iron overload syndromes such as haemochromatosis; treatment with oral iron preparations within 4 weeks before registration; inadequately controlled serum phosphate levels within 4 weeks before registration (any pre-dialysis serum phosphate ≥7.0 mg/dL); any pre-dialysis adjusted serum calcium <8.0 or >11.0 mg/dL within 4 weeks before registration; inadequately controlled Hb levels within 4 weeks before registration (pre-dialysis level <9 or >12 g/dL); malignancy (including haematological malignancy) or previous history of malignancy within 5 years before registration; hepatitis such as chronic hepatitis C or those with severe hepatic dysfunction; and red blood cell transfusion therapy within 12 weeks before registration.

### Study protocol

Patients taking non-iron-based phosphate binders (monotherapy or combination therapy of sevelamer hydrochloride, lanthanum carbonate hydrate, bixalomer, precipitated calcium carbonate) were randomised 1:1 at registration to FC or control (Fig. 1). The registration system assessed eligibility based on the entered information and randomly assigned patients to groups via a permuted block method. In the FC group, non-iron-based phosphate binders were switched to FC at registration. The starting dose was 500 mg (2 tablets of 250 mg of FC containing approximately 60 mg ferric iron per tablet) administered three times daily (1500 mg/day) immediately after meals. Subsequent doses were adjusted weekly as required to maintain serum phosphate at target levels (3.5–6.0 mg/dL) according to the clinical practice guideline for CKD-MBD issued by the JSDT with dose increases of 1500 mg/day up to a maximum of 6000 mg/day. In the FC group, concomitant use of oral calcium preparations was permitted only if the preparation was administered as a calcium supplement at bedtime, and not as a phosphate binder. In the control group, patients continued to take the phosphate binders that they were using before registration, administered according to their prior prescription as defined by their physicians. Concomitant use of drugs other than phosphate binders were permitted and administered at the discretion of patients’ physicians.

Throughout the 24-week study period, serum phosphate was controlled within the range of 3.5–6.0 mg/dL and Hb was controlled within the range of 10–12 g/dL in both groups, according to the guideline for renal anaemia in CKD issued by the JSDT. IV iron was only permitted if iron replacement therapy was required, in the judgment of the patients’ physicians, according to the guideline. For example, if serum ferritin was <100 ng/mL and TSAT <20%. All other oral iron preparations were prohibited in both groups.

### Endpoints

The primary endpoint was change in ESA dose per week from baseline to end of treatment (EOT). The secondary endpoints were cumulative doses of ESA and IV iron from baseline to week 24; ESA dose per week; erythropoiesis resistance index [ERI, defined as ESA dose (IU/week) divided by dry weight (kg)/Hb (g/dL)], red blood cell related parameters [red blood cell count, Hb, Hct, MCV, MCH, and RDW], iron-related parameters (serum iron, serum ferritin, TSAT, hepcidin), serum phosphate and calcium (measured every 4 weeks), and plasma intact FGF 23, C-terminal FGF23, and α-klotho (measured every 12 weeks). The safety endpoints were adverse events and adverse drug reactions.

To calculate total ESA dose across different drug preparations, the doses (µg) of darbepoetin and epoetin beta pegol were converted to IU using the following conversion ratios: epoetin 200 IU = darbepoetin 1 µg = epoetin beta pegol 1 µg^28,29^.

All clinical chemistry analyses were performed by LSI Medience Corporation (Tokyo, Japan) using a standard chemistry autoanalyser. Plasma hepcidin-25 was measured by Hepcidin-25 (Human) ELISA (Peninsula Laboratories International Inc., San Carlos, CA), plasma intact FGF23 was measured by FGF-23 ELISA (Kainos, Tokyo, Japan), plasma C-terminal FGF23 was measured by FGF23 Multi-Matrix ELISA (Biomedica Immunoassays, Vienna, Austria), and plasma α-klotho was measured by Human Soluble α-Klotho Assay (IBL International GmbH, Hamburg, Germany).

### Statistical analysis

We used the full analysis set as the main population for analyses of efficacy. For the primary endpoint, we used the Wilcoxon rank sum test to compare change in ESA dose per week from baseline to EOT. For the secondary endpoints, we used the Wilcoxon rank sum test and analysis of covariance (ANCOVA). Since higher RDW (≥15.5%) was associated with higher risk mortality in patients undergoing haemodialysis^17^, we performed stratified analysis by baseline RDW (<15.5% and ≥15.5%). We calculated the change in RDW and ESA dose from baseline to week 24 in both groups and tested their differences within each stratum using the Wilcoxon rank sum test. Analyses of safety included all participants who received study treatment at least once.

In previous 28-week and 52-week study^28^ of FC in Japanese patients, the mean (SD) change in ESA dose from registration to week 24 was −1045.6 (2579.0) IU. Assuming this change in the FC group and a mean change of zero in the control group, a sample size of 45 participants per group would provide 83.3% power to reject the null hypothesis of no difference between groups by the Wilcoxon rank sum test with a significance level of 5%.

All statistical tests were two-sided with α = 0.05 without adjustment for multiple comparisons, and all analyses were performed using SAS version 9.4 software (SAS Inc., Cary, NC).

## Data Availability

The datasets generated and/or analysed during the study are available from the corresponding author on reasonable request.
